# Cortico-basal oscillations index naturalistic movements during deep brain stimulation

**DOI:** 10.1093/brain/awaf466

**Published:** 2025-12-16

**Authors:** Daryl J Lawrence, Guy Avraham, Jiaang Yao, Lexin Li, Chengchun Shi, Philip A Starr, Simon J Little

**Affiliations:** Joint Graduate Program in Bioengineering, University of California, Berkeley, and University of California, San Francisco, Berkeley, CA 94720, USA; Department of Psychology, University of California, Berkeley, Berkeley, CA 94720, USA; Department of Neurology, University of California, San Francisco, San Francisco, CA 94143, USA; Joint Graduate Program in Bioengineering, University of California, Berkeley, and University of California, San Francisco, Berkeley, CA 94720, USA; Department of Public Health, University of California, Berkeley, Berkeley, CA 94720, USA; Department of Statistics, London School of Economics and Political Science, London WC2A 2AE, UK; Department of Neurosurgery, University of California, San Francisco, San Francisco, CA 94143, USA; Department of Neurology, University of California, San Francisco, San Francisco, CA 94143, USA

**Keywords:** sensorimotor cortex, basal ganglia, intracranial electrode, deep brain stimulation, movement disorder, brain–computer interface

## Abstract

The basal ganglia and sensorimotor cortex are essential nodes of a network that supports motor control. In Parkinson’s disease, disruptions in this network lead to rigidity and slowness during movement execution. Deep brain stimulation (DBS) of the basal ganglia has proved effective in alleviating Parkinson’s disease-related hypokinetic symptoms, and sensing-enabled neurostimulators now afford the opportunity to detect cortico-basal oscillations during motion. However, the specific contributions of these motor network nodes to chronic, naturalistic movement and the effects of DBS on circuit dynamics are not well understood.

To address these gaps, we recorded >530 h of cortical and subcortical signals from 15 Parkinson’s disease patients (27 hemispheres) during unsupervised, unconstrained daily activities and subthalamic or pallidal DBS. Synchronized wrist-worn accelerometers tracked forearm speeds, supporting the evaluation of neural biomarkers related to motion. Our study validated and extended the known relationship between cortical and subcortical beta power (13–30 Hz) and movement. We showed that cortical low (13–20 Hz) and high (21–30 Hz) beta movement-related desynchronization effectively distinguished between mobile and stationary states. In the subthalamic nucleus and globus pallidus interna, high beta movement-related desynchronization and gamma (40–80 Hz) movement-related synchronization exhibited significant group-level correlations with movement kinematics. When stimulated at 130 Hz, cortical stimulation-entrained gamma oscillations at the half-harmonic (∼65 Hz) were observed. Furthermore, cortical entrained gamma movement-related synchronization was a stronger predictor of motion than broadband gamma movement-related synchronization.

We developed machine learning models to predict naturalistic movement over extended periods using spectral features from brief neural recordings (0.5–8 s epochs). Cortical models outperformed subcortical models, although combining cortico-basal signals yielded the highest model performance (area under the curve > 0.85 for binary movement state classifiers; Pearson’s *r* statistic > 0.68 for continuous forearm speed regressors). Higher DBS current amplitudes were associated with reduced beta movement-related desynchronization and low gamma (40–60 Hz) movement-related synchronization in the subthalamic nucleus and globus pallidus interna. This negatively impacted the accuracy of the subcortical models, whereas cortical and cortico-basal model performance remained stable across stimulation amplitudes.

Our study demonstrates that cortico-basal nodes of the motor network encode complementary kinematic information, which can be integrated to enhance the accuracy and stability of chronic, naturalistic movement decoding during deep brain stimulation. These insights support the development and integration of therapeutic brain–computer interfaces with closed-loop, adaptive DBS to leverage rapid and precise movement-predictive models for the treatment of motor network disorders.

## Teaser

By recording over 530 hours of intracranial and accelerometry signals in patients with Parkinson’s disease, Lawrence *et al.* show that the sensorimotor cortex and basal ganglia contain complementary motor information. Integrating these signals enhances the decoding of naturalistic movements during deep brain stimulation.

## Introduction

Precise motor control is essential for executing autonomous and volitional movements.^1^ The basal ganglia and sensorimotor cortex are integral nodes of the motor network that operate together to enable accurate, well-coordinated movements.^2,3^ Within the basal ganglia circuitry, the subthalamic nucleus (STN) and globus pallidus internus (GPi) regulate movement by selecting amongst competing motor programmes and relaying information to the cortex.^4-6^ The sensorimotor cortex is generally responsible for planning and executing voluntary movements via neuronal projections to the brainstem and spinal cord.^7,8^ The basal ganglia and sensorimotor cortex might provide complementary information and perform distinct roles in coordinating motor activity, although the specific neural dynamics underlying this relationship are not yet well established.^9,10^

Laboratory-based recordings of local field potentials from the basal ganglia and sensorimotor cortex have identified potential neurophysiological biomarkers linked to kinematic features.^11,12^ In the STN, a reduction in beta power (12–30 Hz) and an increase in broadband gamma power (55–90 Hz) are associated with elevated motor vigour in constrained, ballistic motor tasks.^13-16^ In the sensorimotor cortex, a decrease in alpha and beta power, along with an increase in broadband gamma power, have been observed during motor planning and execution.^17,18^ Although these findings provide insights into movement-related spectral changes, they are based primarily on neural data recorded during brief, supervised, highly controlled tasks in perioperative settings. Reliable long-term intracranial decoding of human motion, in naturalistic environments over extended periods, has not yet been achieved. Identifying the cortical and subcortical biomarkers of real-time movement is essential for understanding the specific contributions of each node of the motor network to the execution of selected motor plans.^19^

Impairment of the motor network leads to movement disorders, such as Parkinson’s disease and essential tremor, which are debilitating and often challenging to treat.^20^ Although open-loop deep brain stimulation (DBS) has proved effective, particularly for Parkinson’s disease, its efficacy is limited by fluctuations in symptom severity and medication levels.^21^ New technological advancements, including brain–computer interfaces (BCI) and closed-loop, adaptive deep brain stimulation (aDBS), present promising opportunities for developing personalized treatments with greater therapeutic benefit. Historically, these technologies have been developed independently, with BCIs focusing on precise neural signal decoding and aDBS targeting clinically defined states (e.g. fluctuations in dopaminergic medication levels) or underlying physiology (e.g. beta bursts).^22,23^ However, given the core deficit of movement disorders, we investigate an approach that integrates these techniques, BCI–aDBS. The main concept here is to detect a patient’s intention to move from intracranial brain signals and rapidly ramp up DBS at that moment to support motor execution.

Recently, our group demonstrated the proof of principle of this BCI–aDBS method in a single Parkinson’s disease patient.^24^ We developed aDBS policies that targeted movement during brief, constrained motor tasks. That study demonstrated accurate cortically based movement decoding and improved hand speeds during a keyboard typing task, with a reduction in involuntary, dyskinetic movements during rest. However, the neural biomarkers of continuous, unconstrained movement remain undetermined, hindering the development of personalized, movement-responsive BCI–aDBS therapies for disorders of movement at scale.^25^

A new generation of neurostimulators for DBS have been developed that can record neural activity while delivering electrical current to the basal ganglia.^22,26^ These recordings can be collected in naturalistic environments, providing an ideal platform to investigate the complementary roles of the basal ganglia and cortex in movement. We recorded 539 h of cortical and subcortical signals from a cohort of Parkinson’s disease patients (15 patients; 27 independent hemispheres) implanted with neurostimulators while they performed unconstrained activities of daily living at home during therapeutic DBS. Wrist-worn accelerometers were used to track forearm speeds of patients and synchronized to neural recordings.^27,28^ We identified site-specific neural signals related to motion and developed machine learning (ML) models for movement prediction. Furthermore, we leveraged these findings to elucidate the effects of varying the stimulation amplitude on movement decoding, ML performance and site-specific kinematic biomarkers.

## Materials and methods

### Participant selection and assessment

We enrolled 15 individuals [mean ± standard error of the mean (SEM) age: 63 ± 3 years] with idiopathic Parkinson’s disease from the movement disorders surgery clinic at the University of California, San Francisco (UCSF) in accordance with the Declaration of Helsinki (Table 1). The study was reviewed by the UCSF Institutional Review Board (ClinicalTrials.gov: NCT03582891) under an investigational device exemption (G180097/R003) for the Summit RC+S device (Medtronic, Inc.).^21^ These patients presented with standard clinical indications for STN (12 patients) or GPi (three patients) DBS as confirmed by a movement disorders neurologist following the criteria outlined by the Movement Disorders Society for Parkinson’s disease diagnosis. The motor function of the patients prior to implantation was assessed using the Unified Parkinson’s disease Rating Scale (UPDRS) Part III, and their cognitive abilities were evaluated through the Montreal Cognitive Assessment. Participants with a Montreal Cognitive Assessment score of ≤20, or those with an untreated mood disorder as determined by a neuropsychologist, were excluded from the study.

**Table 1:** Patient demographics, stimulation settings and clinical information

Patient ID	Age, years; sex	Dx, years	Stimulation target	Hemisphere	Stimulation frequency, Hz	Stimulation level, mA	UPDRS-III		Carbidopa–levodopa (daily dosage)
							OFF	ON	
Pat-01	60; M	13	STN	L	130	2.2–2.8	49	5	25–100 mg IR (5 times daily)
				R	130	2.7–3.5			
Pat-02	69; M	25	STN	L	130	1.9–2.5	45	22	50–200 mg CR (4 times daily)
				R	130	1.4–1.6			
Pat-03	46; M	10	STN	L	130	1.5–2.2	41	10	25–100 mg IR (2 times daily)
				R	130	1.5–2.2			
Pat-04	63; M	18	STN	L	130	2.9	44	11	25–100 mg IR (5 times daily)
				R	130	1.9–2.0			
Pat-05	76; M	13	STN	L	130	1.9–2.2	29	12	25–100 mg IR (4 times daily)
				R	130	1.0			
Pat-06	65; M	11	STN	L	130	0.0–2.9	35	12	24–95 mg ER (4 times daily)
				R	130	0.0–3.1			
Pat-07	48; M	17	STN	L	130	0.0–1.9	32	5	24–95 mg IR (5 times daily)
				R	130	0.0–1.9			
Pat-08	69; M	14	STN	L	130	0.0–2.6	30	7	24–95 mg ER (5 times daily)
				R	130	0.0–2.4			
Pat-09	62; M	16	STN	L	130	0.0	34	9	25–100 mg IR (5 times daily)
				R	130	0.0			
Pat-10	75; M	19	STN	L	130	0.0	31	10	49–195 mg ER (4 times daily)
				R	130	0.0			
Pat-11	79; M	14	STN	L	159	3.4	37	24	36–145 mg ER (4 times daily)
Pat-12	34; F	18	STN	R	167	1.2–2.2	61	16	25–100 mg IR (5 times daily)
Pat-13	53; M	14	GPi	L	130	0.0–1.1	49	19	49–195 mg ER (4 times daily)
				R	130	0.0–1.6			
Pat-14	71; M	15	GPi	L	179	3.7	66	24	25–100 mg IR (4 times daily)
				R	179	2.8			
Pat-15	69; M	10	GPi	L	149, 188	0.0–3.9	31	15	25–100 mg IR (5 times daily)

CR = controlled release; Dx = disease duration; ER = extended release; F = female; GPi = globus pallidus internus; IR = immediate release L = left hemisphere; M = male; MFG = medial frontal gyrus; PoG = post-central gyrus; PrG = pre-central gyrus; R = right hemisphere; SFG = superior frontal gyrus; SPL = superior parietal lobe; STN = subthalamic nucleus; UPDRS = Unified Parkinson’s Disease Rating Scale.

### Surgical implantation

Quadripolar depth leads were inserted surgically into either the STN (Medtronic 3389 lead) or the GPi (Medtronic 3387 lead).^29^ Electrocorticography strips, designed solely for sensing purposes, were positioned along a parasagittal trajectory to ensure that at least one contact aligned with both the precentral and the postcentral gyri.^22^ The precise locations of these electrodes were determined intraoperatively using cone beam CT fused to preoperative MRI scans. The cortical and subcortical leads were connected to a Summit RC+S implantable pulse generator (model B35300R) above the ipsilateral pectoralis muscle via 60 cm lead extenders (model 37087).^30^

### Intracranial data acquisition and preprocessing

**Figure 1:**
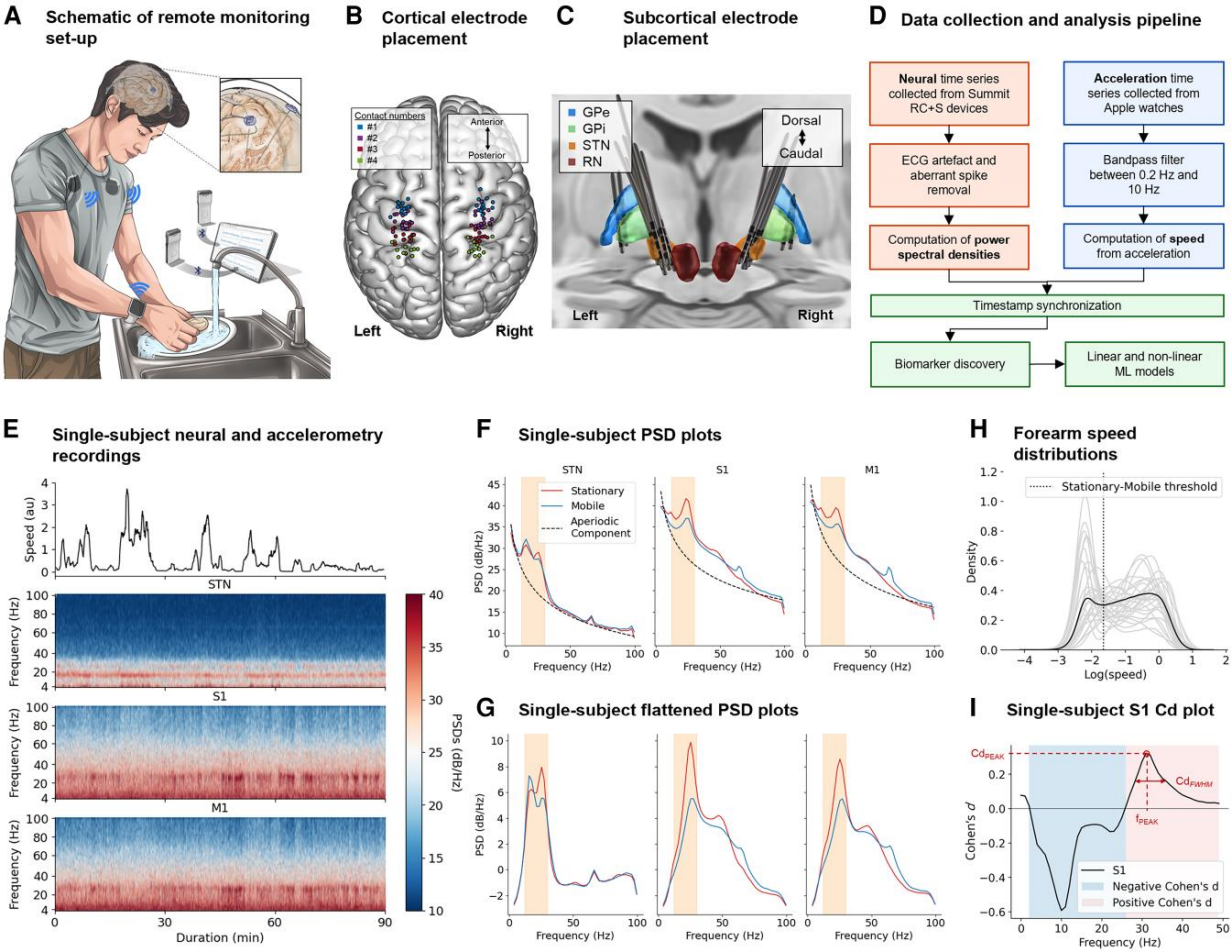
**Anatomical localization of implanted electrodes and single-subject example of processed neural and accelerometry data.** (**A**) Schematic diagram of a patient performing an activity of daily living while streaming data from implanted neurostimulators and wrist-worn accelerometers. (**B**) Cortical electrodes represented on a template brain. Five of the 15 patients in our cohort used an overlapping sensing configuration (electrode pairs: 1–3 and 2–4). The remaining 10 subjects used a non-overlapping configuration (electrode pairs: 1–2 and 3–4). (**C**) Subcortical electrodes represented on a template brain in the globus pallidus externus (GPe), globus pallidus internus (GPi), subthalamic nucleus (STN) and red nucleus (RN). (**D**) Neural and accelerometry data processing pipeline. (**E**) Single-subject (Pat-01) spectrograms based on data streamed from the left STN, primary somatosensory cortex (S1) and primary motor cortex (M1). Right forearm speeds were recorded simultaneously. (**F**) Single-subject average power spectral densities (PSDs) were computed from the STN, S1 and M1 signals and fitted with the spectral parameterization algorithm to make comparisons between mobile and stationary states. (**G**) The corresponding flattened PSD plots are displayed with the beta band (12–30 Hz) highlighted. (**H**) Forearm speeds from all patients in our cohort were combined and displayed in a kernel density estimate plot. A local minimum was identified at the 28th percentile of the distribution (termed the movement state threshold) and used to distinguish between mobile and stationary states. (**I**) Cohen’s *d* (Cd) effect sizes based on the flattened PSDs from a single subject were calculated and used to create a Cd plot. These values quantified the differences in spectral power between movement states. The frequencies with positive Cd values, indicating an increase in spectral power during mobile states compared with stationary states, are highlighted in red. Several characteristics, such as the peak frequency (*f*_PEAK_), maximum Cohen’s *d* (Cd_PEAK_) and full width at half maximum (Cd_FWHM_), were extracted from this band. The frequencies with negative Cd values are highlighted in blue. FWHM = full width at half maximum.

We collected >530 h of neural data while patients performed unsupervised, unconstrained activities of daily living at home (May 2020–May 2023) (Fig. 1A).^22,31^ Baseline recordings were performed in 7 of the 15 patients at a stimulation amplitude of 0 mA, prior to the initiation of DBS therapy, to establish the initial motor symptom profiles of the patients. As part of clinical care, a movement disorders specialist identified the optimal DBS electrode contacts and stimulation amplitude to maximize therapeutic benefit while minimizing side effects, such as dyskinesia. Eleven patients were recorded at various stimulation amplitudes during the DBS parameter optimization phase.

Throughout the study, participants continued to take their prescribed antiparkinsonian medications according to clinical guidance from their treating neurologist. Electrocorticography leads captured neurophysiological data from the primary motor (M1) and somatosensory (S1) cortices, while depth leads recorded signals from the STN or GPi. The precise locations of these cortical and subcortical electrodes were reconstructed using the Locate Electrodes Graphical User Interface toolbox (Fig. 1B) and Lead-DBS toolbox (Fig. 1C), respectively. The neural data were recorded at a sampling frequency of 250 or 500 Hz and transmitted from the RC+S neurostimulator devices to a nearby telemetry module, then to a Microsoft Windows tablet. The tablet was equipped with custom software, built on the Summit RC+S application, developed in compliance with US Food and Drug Administration regulations (CFR 820.30) and available at https://github.com/openmind-consortium.

Cortical and subcortical local field potentials recorded by the RC+S devices were initially preprocessed in MATLAB R2022b using the Analysis-rcs-data toolbox (Fig. 1D).^32^ They were then filtered from 0.8 to 100 Hz with an infinite impulse response elliptic bandpass filter (1 dB passband ripple; 100 dB attenuation). For artefact detection and removal, we squared the magnitude of the neural signals and performed Gaussian smoothing over a moving 1 s window. Amplitudes exceeding five times the median were identified as aberrant spikes, and the time series corresponding to these periods were removed. Removal of ECG artefacts was carried out using two MATLAB libraries, Perceive and PerceptHammer.^33,34^ Perceive detected QRS-like patterns within 10 min intervals (to accommodate variations in ECG artefacts over time).^33^ These patterns were then averaged within each session and used as initializations for the template subtraction pipeline from the PerceptHammer library.^34^ The initial template facilitated the identification of ECG artefact locations within the underlying signal, and this template was updated continuously using Woody’s adaptive filter. However, this recursive process of artefact identification and template updating had the potential to transform the template into one that matched non-artefactual low-frequency spectral power, leading to inaccurate template subtraction. To mitigate this issue, we constrained the transformation of the initial template by comparing the updated and initial templates via normalized cross-correlation. If the cross-correlation between templates fell below a threshold of 0.9, the initial template was used for the entire artefact identification and subtraction process without iterative modification by the adaptive filter.

### Accelerometry data acquisition and preprocessing

Participants wore an Apple watch (Apple Inc.) on the wrist contralateral to the hemisphere where the RC+S device was implanted. For patients with bilateral implants, two watches were worn to record movement in both forearms simultaneously at a sampling rate of 50 Hz.^24^ Accelerometry data were transmitted from the watches to nearby iPhones through the StrivePD iOS application (Rune Labs Inc.). Previously validated external algorithms generated tremor and dyskinesia scores at 1 min intervals.^35^ Accelerometry signals in the *x*, *y* and *z* directions were integrated to derive the respective velocity measurements, filtered between 0.2 and 10 Hz using a fourth-order Butterworth bandpass filter. The resulting *x*, *y* and *z* velocities were used to compute absolute forearm speeds.

### Statistics

For our statistical analyses, we used various Python (v.3.9.17) packages, including SciPy and Statsmodels. One-way ANOVAs were used to explore significant differences between the means of two or more groups. Wilcoxon signed-rank tests were used for the non-parametric comparison of paired samples. All relevant *P*-values underwent correction for multiple comparisons through the false discovery rate (FDR) procedure.^36^ Linear mixed models (LMMs) were constructed using the Pymer4 library, with individual patients and hemispheres considered as random effects. These LMMs were used to examine the influence of stimulation current amplitude on neural biomarkers and the performance of movement-predictive models.

### Power spectral analyses

Cortical and subcortical power spectra were computed from the preprocessed local field potentials using a one-dimensional Fourier transform in the NumPy package (numpy.fft.fft) (Fig. 1E). The neural signals were divided into non-overlapping 0.5 s epochs, and a Hann window was applied to calculate power spectral densities (PSDs) ranging from 0 to 100 Hz and spanning 2 Hz each (Fig. 1F). To eliminate the aperiodic component in each power spectrum, the spectral parameterization (SpecParam) algorithm was used across the 4–100 Hz range (Fig. 1G).^37,38^ To minimize the impact of involuntary movements on our analyses, we excluded epochs that had a non-zero tremor or dyskinesia severity score, because these scores indicated the presence of symptoms of significant severity.^35^ The RC+S and Apple watch devices were set to computer clock time based on the network time protocol to synchronize the time stamps from both devices. As an added step, we identified the time lag between their clocks that maximized the cross-correlation of their respective accelerometry measurements.^39^ Once the time stamps were aligned, we linearly interpolated the wearable accelerometry data to determine the absolute forearm speed for each 0.5 s segment.

We combined forearm speed measurements from all patients to produce a group-level distribution and identified a local minimum to be used as a standardized threshold for distinguishing between periods of movement (mobile states) and rest (stationary states) (Fig. 1H). We labelled epochs based on their movement states and calculated Cohen’s *d* (Cd) effect sizes using their PSDs, adjusting for class imbalances with pooled standard deviations (SDs) (Fig. 1I).^40^ The Cd values provided a signed, normalized metric to quantify the discriminative differences between the flattened PSDs in the mobile or stationary states. Additionally, we computed Spearman’s ρ, a non-parametric measure that is robust to outliers, to examine the correlation between the PSDs from each brain region and the contralateral absolute forearm speed measurements.

### Machine learning model development and evaluation

We developed linear and non-linear classifiers and regressors using the Python-based scikit-learn (sklearn) library to distinguish between mobile and stationary states or predict absolute forearm speeds, respectively. Prior to model training, we standardized the data by removing the mean and scaling it to unit variance. To evaluate the performance of the classifiers, we used the area under the receiver operating characteristic curve (AUC) as our primary metric. The AUC is robust to class imbalance, making it particularly effective for evaluating model performance across various decision thresholds.^41^  ^,42^ Moreover, we calculated accuracies, F1-scores and positive predictive values, as secondary metrics. To balance our training datasets, we used the synthetic minority over-sampling technique (SMOTE).^43^ For the regression analysis, we assessed model performance using Pearson’s correlation coefficient (*r* statistic) to compare true and predicted speed values.^44^ As a secondary metric, we used the mean squared error.^45^

To reduce both multicollinearity and complexity, we calculated six canonical power bands (PBs) to serve as features for our linear models: alpha (8–12 Hz), low beta (13–20 Hz), high beta (21–30 Hz), low gamma (40–60 Hz), stimulation-entrained gamma (63–67 Hz) and high gamma (70–90 Hz). In addition, we used principal component analysis to generate subject-specific features. This approach enabled a direct comparison between the effectiveness of canonical PBs versus personalized principal components (PCs) for predicting movement. For more complex non-linear models, we used the complete set of PSDs as features, without any dimensionality reduction.

For the classifiers, we shuffled and stratified our data based on mobile and stationary state labels before splitting the data into training (80%) and test (20%) sets. This ensured that both sets preserved the class distribution of the original dataset, preventing folds from having missing classes or imbalanced proportions, which could lead to biased model evaluation. Recording sessions were generally conducted on different days, including varying stimulation levels owing to active titration by both patients and clinicians. Therefore, stratification also ensured that the training and test sets had samples from various days and stimulation amplitudes. In subsequent sections, we assessed the performance of models when transferred across these different sessions and stimulation levels. For the regressors, stratification was not used because we were predicting continuous forearm speeds.

To determine the specific contributions of each feature to model performance, we calculated the permutation feature importance for each PB and brain region.^46^ This process involved shuffling the sample values (10 000 permutations) to diminish the predictive power of each feature, allowing us to measure the change in model performance when predictions were made using the altered dataset. We also computed the conditional mutual information between each PB and forearm speed measurements, and between PBs in each brain region and movement speeds.^47^ This analysis quantified the reduction in uncertainty about forearm motion attributable to each PB and brain region, while accounting for the contribution of movement-related information from other PBs.^48,49^ To examine the effects of the varying DBS current amplitudes on kinematic biomarkers, we computed movement-related Cd effect sizes for each canonical power band and brain region at each stimulation level. Additionally, we explored the influence of the time interval between sessions on model performance. This was done by training models with samples from one session and testing them with data from another session at different time points. We also evaluated how stimulation amplitudes influenced our ability to decode movement by using 5-fold stratified cross-validation to measure model performance at each stimulation level. We quantified the changes in Cd values and model AUCs across different stimulation levels using LMMs.

## Results

### Cortical and subcortical biomarkers of naturalistic movement

We recorded intracranial signals from the S1 and M1, and from the STN (22 hemispheres) or GPi (five hemispheres), during DBS in patients with Parkinson’s disease (mean ± SEM duration of data collected per hemisphere: 4.2 ± 0.3 h). A total of 539 h of data were collected, comprising 213 h at 0 mA and 326 h at stimulation amplitudes of >0 mA. Participants used wrist-worn accelerometers (Apple watches) to record their forearm speeds during unconstrained everyday activities. The time stamps of the RC+S and Apple watches were synchronized to correct for any time lags (mean ±SEM absolute time lag before correction: 0.6 ± 0.2 s). Commencing with the raw local field potentials, we computed flattened PSDs within 500 ms epochs. Of these epochs, 65% (353 h; 227 h at stimulation amplitudes of >0 mA and 126 h at 0 mA) were free from tremor or dyskinesia based on Apple watch scores. Using the local minimum of the group-level bimodal combined forearm speed distribution (28th percentile), we distinguished between mobile and stationary states.

**Figure 2:**
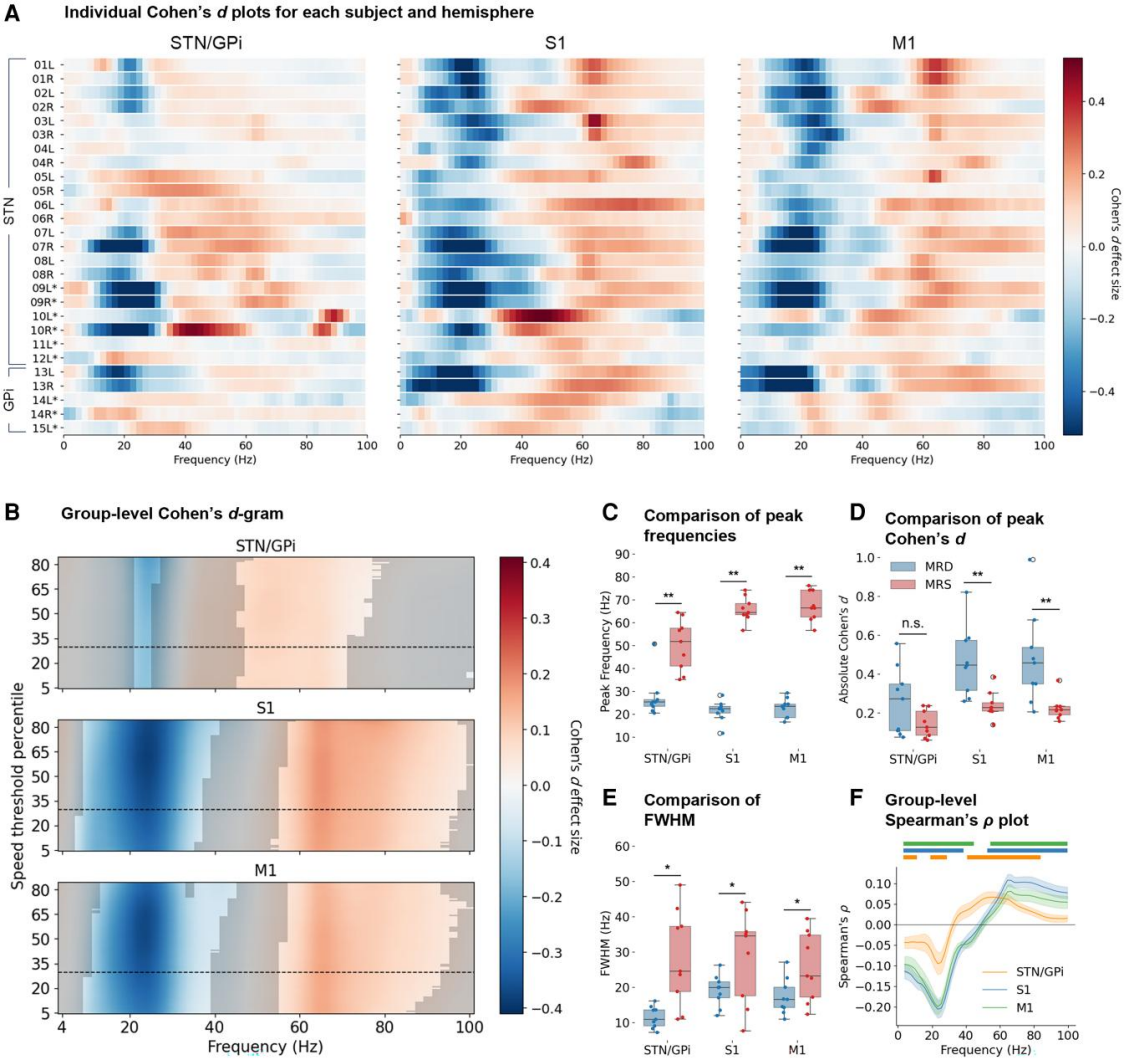
**Cortical and subcortical biomarkers of naturalistic movement.** (**A**) Cohen’s *d* (Cd) plots for each patient and hemisphere were computed using STN/GPi (*left*), S1 (*middle*) and M1 (*right*) signals. Four of 15 patients in our cohort had a different stimulation frequency from 130 Hz and two were recording while stimulation was OFF (marked by asterisks). The largest frequency range with contiguous positive Cd values (highlighted in red) was termed the movement-related synchronization (MRS) band, whereas that with contiguous negative Cd values (highlighted in blue) was termed the movement-related desynchronization (MRD) band. In cases where a hemisphere exhibited multiple MRD or MRS bands, the band with the highest average absolute Cd value was selected for further analysis. (**B**) Each percentile (between the 5th and 95th percentiles) of the group-level forearm speed distribution (from Fig. 1H) was used as a threshold to produce one-dimensional Cd plots discriminating slower from faster movements. These plots were concatenated to create two-dimensional Cohen’s ‘*d*-grams’ (Cd-grams). The horizontal dotted line in each Cd-gram lies on the 28th percentile, which distinguishes between mobile and stationary states. The grey masks indicate the regions that did not survive false discovery rate (FDR) correction (*P* ≥ 0.05). (**C**) Using the 28th percentile threshold, we identified the frequencies with the lowest (most negative) and highest (most positive) Cd values from each hemisphere; these were termed the MRD and MRS Cd_PEAK_ frequencies, respectively. (**D** and **E**) Additionally, we calculated the absolute Cd_PEAK_ magnitudes (**D**) and the full widths at half maximum (Cd_FWHM_) (**E**) of the MRD and MRS bands (based on the Cd plots in Fig. 2A). (**F**) FDR-corrected average Spearman correlation (ρ) values between the power spectral densities and the absolute forearm speeds were computed. The resulting significant frequency bands are highlighted by the colour-coded bars above the respective plots. *P ≤ 0.05, **P ≤ 0.01 and ***P ≤ 0.001. FWHM = full width at half maximum; GPi = globus pallidus internus; M1 = primary motor cortex; S1 = primary somatosensory cortex; STN = subthalamic nucleus.

Based on this distinction between movement states, we calculated Cd effect sizes for frequencies ranging from 0 to 100 Hz for each subject and hemisphere (Fig. 2A). Negative and positive Cd values indicated a decrease and increase in spectral power, respectively, during the mobile state compared with the stationary state.^50^ Frequency ranges with contiguous PSDs showing negative Cd values were identified as movement-related desynchronization (MRD) bands, whereas those with positive Cd values were identified as movement-related synchronization (MRS) bands.^12-17^ For our main analyses, we opted to focus on the subset of hemispheres that were stimulated at 130 Hz (nine patients; 16 STNs and 2 GPis), to avoid interference from varying stimulation frequencies. Additionally, analysis with all data, irrespective of stimulation frequency, was also completed and is included in the Supplementary material.

Shifting the speed threshold between the 5th and 95th percentiles of the combined forearm speed distribution, we computed the average Cohen’s ‘*d*-gram’ for each site (Fig. 2B). This represented the movement-discriminative ability of each PSD (within 0–100 Hz) across a range of immobile/slower versus faster distributional splits. Significant MRD was observed in the alpha, low beta and high beta ranges in cortical areas. In contrast, in the STN/GPi, high beta MRD, but not low beta MRD, reached significance at the group level (FDR-corrected, *P <* 0.05). However, on an individual level, six patients (nine STNs and two GPis) also demonstrated significant MRD in the low beta frequency range, underscoring the variability in the predictive power of canonical PBs across participants.

Significant MRS was found in the 40–80 Hz range for the STN/GPi and in the 60–100 Hz range for the S1 and M1 (FDR-corrected *P <* 0.05). These findings revealed significant predictive power from both cortical and subcortical regions, albeit within site-specific frequency ranges.^12,19,50^ To assess the robustness of these findings across different dopaminergic medication levels, we recreated the Cohen’s *d*-grams within 15 min intervals, during which variations in medication levels were likely to be minimal, and averaged them (FDR-corrected *P <* 0.05; Supplementary Fig. 1A). We also calculated the average group-level Cohen’s *d*-gram using the complete dataset, including periods with tremor or dyskinesia (Supplementary Fig. 1B). These group-level Cohen’s *d*-grams revealed consistent alpha/beta MRD and gamma MRS bands, despite variations in medication level over time or analysis of the full recording duration.^51^

We extracted several characteristics from the MRD and MRS bands in each Cd plot for comparison, including the peaks in Cd (Cd_PEAK_) and their respective frequencies (*f*_PEAK_). Specifically, we determined the highest Cd value in the MRS band (MRS Cd_PEAK_) and the most negative Cd value in the MRD band (MRD Cd_PEAK_). There was no significant difference in the MRD Cd_PEAK_ frequencies of cortical versus subcortical sites (median ± SEM peak frequencies; STN/GPi, 24 ± 4 Hz; S1, 22 ± 1 Hz; M1, 23 ± 1 Hz; one-way ANOVA: *F =* 2.18, *P =* 0.13; Fig. 2C). However, cortical MRS Cd_PEAK_ frequencies were higher than those in the STN/GPi (STN/GPi, 52 ± 4 Hz; S1, 64 ± 2 Hz; M1, 66 ± 2 Hz; *F =* 12.16, *P <* 10^−4^; *post hoc* one-sided Wilcoxon signed rank test: *W =* 0.0, *P <* 0.002). Cortical MRS Cd_PEAK_ values were commonly ∼65 Hz, suggesting a link between stimulation-entrained gamma power and volitional movement.^19,21^

We also compared the MRD and MRS Cd_PEAK_ magnitudes in each brain region. Cortical beta MRD Cd_PEAK_ demonstrated a greater magnitude than cortical gamma MRS Cd_PEAK_ (*W =* 45.0, *P =* 0.003; Fig. 2D). Additionally, cortical MRD Cd_PEAK_ (*W =* 0.0, *P =* 0.002) and MRS Cd_PEAK_ (*W =* 6.0, *P =* 0.03) exhibited higher magnitudes than those observed in the STN/GPi, indicating that cortical biomarkers were more predictive of naturalistic motion.^19,24^ Moreover, we calculated the full width at half maximum of the Cd values (Cd_FWHM_) within each MRD and MRS band. The MRS Cd_FWHM_ was significantly wider than the MRD Cd_FWHM_ at each site (STN/GPi: *W =* 2.0, *P =* 0.02; S1: *W =* 5.0, *P =* 0.03; M1: *W =* 8.0, *P =* 0.049; Fig. 2E). To examine further the predictive capabilities of each brain region in estimating continuous forearm speed values, Spearman correlations between each PSD and absolute forearm speed were computed (Fig. 2F). We found that alpha MRD, beta MRD and gamma MRS encoded continuous forearm speeds in each brain region (FDR-corrected *P <* 0.05). These results emphasized the presence of site-specific biomarkers associated with naturalistic motion.^8^

### Movement decoding using multivariate signals and machine learning

**Figure 3:**
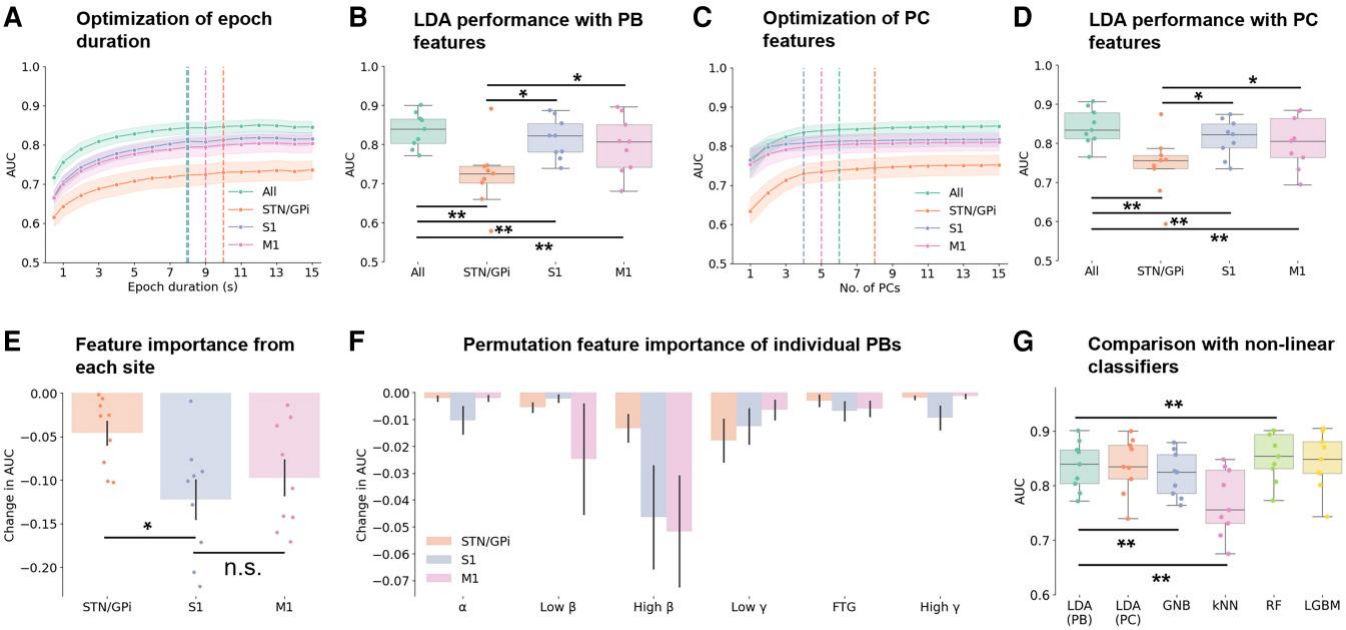
**Evaluating the performance of binary movement state classifiers.** We performed hyperparameter tuning on linear discriminant analysis (LDA) classifiers using canonical power band (PB) features. (**A**) The optimal epoch durations were identified for single-site (STN/GPi, S1 or M1) and combined models using the area under the receiver operating characteristic curve (AUC). (**B**) These models were then tested on a holdout set for each patient. We also evaluated the performance of classifiers trained on personalized features. (**C**) We performed principal component analysis and identified the optimal number of principal component (PC) features for each classifier. (**D**) The AUCs of the personalized classifiers with PC features were computed on the holdout set. (**E** and **F**) Permutation feature importance was assessed to quantify the unique contributions of each site (STN/GPi, S1 or M1) (**E**) and canonical PB (**F**) to the performance of the combined model. (**G**) Additional linear and non-linear models [Gaussian naïve Bayes (GNB), *k*-nearest neighbours (KNN), random forests (RF) and light gradient boosted machines (LGBMs)] were developed using all power spectral density features from each site, to avoid restricting the models to the six canonical PB features. *P ≤ 0.05 and **P ≤ 0.01. FTG = finely tuned gamma; GPi = globus pallidus internus; M1 = primary motor cortex; S1 = primary somatosensory cortex; STN = subthalamic nucleus.

Converging evidence indicates that the nervous system encodes movement-related neural activity across several frequency ranges simultaneously.^19,52^ This suggests that combining neural features can potentially enhance the capacity to decode forearm movements. Therefore, we developed ML models with multiple features to classify stationary and mobile states or predict continuous forearm speeds during unconstrained, naturalistic motion. These models were developed using canonical PBs from an individual site (STN/GPi, S1 or M1) or by combining PB features from all three regions. Using linear discriminant analysis and 5-fold cross-validation on the training dataset, we identified the optimal epoch durations for each region by comparing the AUC scores (STN/GPi, 8 s; S1, 10 s; M1, 8 s; and combined, 9 s; Fig. 3A). These represented the epoch durations above which there was no improvement in model performance by >0.01.

Significant differences in AUC between single-site and combined classifiers were observed on the holdout dataset (*F =* 5.35, *P =* 0.004; Fig. 3B). The combined classifier had the highest performance (mean ± SEM AUC: 0.84 ± 0.01; *W* > 42.0, *P <* 0.002).^22^ Cortical sites were more predictive of movement states compared with the STN/GPi (*W* > 40.0, *P <* 0.03), although there was no significant difference in performance between the S1 and M1 models (*W =* 24.0, *P =* 0.46).^53,54^ To provide a comprehensive assessment of our models, we also computed several other metrics, such as balanced accuracy, F1 score, sensitivity, specificity and positive predictive value (Table 2).

**Table 2:** Average group-level performance of linear models from each brain region

Metric	Combined	STN/GPi	S1	M1
Classifier performance, mean ± SEM				
AUC	0.84 ± 0.01	0.72 ± 0.02	0.80 ± 0.02	0.80 ± 0.02
Balanced accuracy	0.76 ± 0.01	0.66 ± 0.02	0.73 ± 0.01	0.73 ± 0.02
F1 Score	0.80 ± 0.02	0.71 ± 0.02	0.77 ± 0.02	0.78 ± 0.02
PPV	0.86 ± 0.03	0.79 ± 0.04	0.84 ± 0.03	0.84 ± 0.03
Sensitivity	0.75 ± 0.02	0.66 ± 0.02	0.72 ± 0.02	0.73 ± 0.02
Specificity	0.77 ± 0.01	0.67 ± 0.02	0.74 ± 0.01	0.74 ± 0.02
Regressor performance, mean ± SEM				
*r* statistic	0.64 ± 0.03	0.41 ± 0.04	0.58 ± 0.03	0.58 ± 0.04
MSE	0.41 ± 0.02	0.57 ± 0.04	0.48 ± 0.03	0.48 ± 0.02

AUC = area under receiver operating characteristic curve; GPi = globus pallidus internus; M1 = primary motor cortex; MSE = mean squared error; PPV = positive predictive value; *r* statistic = Pearson’s correlation coefficient between true and predicted values; S1 = primary somatosensory cortex; SEM = standard error of the mean; STN = subthalamic nucleus.

Prior studies have demonstrated that customizing features for individual patients can enhance the performance of predictive models.^22,28^ Consequently, besides using canonical power bands, we used principal component analysis and identified the number of PC features for optimal classifier performance (STN/GPi, eight; S1, four; M1, five; combined, six; Fig. 3C). We also computed the cumulative explained variance of these PCs (mean ± SEM variance: STN/GPi, 81% ± 2%; S1, 59% ± 2%; M1, 65% ± 2%; combined, 47% ±2%; Supplementary Fig. 2A) and the PC loadings of the first PC (FDR-corrected *P <* 0.05; Supplementary Fig. 2B). As with canonical PBs, the combined classifiers yielded the highest AUC (mean ±SEM AUC: 0.83 ± 0.01; *F =* 3.89, *P =* 0.02; *W =* 45.0, *P =* 0.002; Fig. 3D). We found no significant difference between linear models using personalized PCs and those using canonical PBs (*W =* 20.0, *P =*0.63). This was probably attributable to the use of six canonical PB features per site. However, real-world devices might impose additional model restrictions (e.g. maximum of four PBs for the Summit RC+S), which might lead to improved performance through personalization of features.^24^

We determined the permutation feature importance of each site to quantify their specific contributions to movement decoding (mean ± SEM change in AUC: STN/GPi, −0.05 ± 0.01; S1, −0.12 ± 0.02; M1, −0.10 ± 0.02; *W =* 0.0, *P =* 0.004; Fig. 3E). The contribution from S1 was greater than that from the STN/GPi (*W =* 41.0, *P =* 0.041) but similar to that from M1 (*W =* 19.0, *P =* 0.37). These findings suggested that the basal ganglia and sensorimotor cortex encode distinct, non-redundant movement-related information.^7,8,54^ We examined the permutation feature importance of individual PBs in each brain region. Among these, cortical high beta power demonstrated the highest feature importance (mean ± SEM change in AUC: −0.10 ± 0.02), indicating that it was the most predictive biomarker of naturalistic movement amongst the PBs (*F =* 2.74, *P =* 0.0005; *W =* 6.0, *P =* 0.027; Fig. 3F).^14^

Although simple linear classifiers can be embedded on current implantable neurostimulators, more advanced models might yield higher performance for real-time movement decoding.^23^ Therefore, we evaluated the effectiveness of more complex models, including *k*-nearest neighbour (kNN), random forest (RF) and light gradient-boosting machine (LGBM) (Fig. 3G). We also included all 50 PSDs from each site as features for these non-linear models, rather than restricting them to the canonical PB features. The RF classifier achieved a small but significant improvement over linear models that used PB features (mean ± SEM AUC: 0.85 ± 0.01), with a difference in AUC of 0.016 ± 0.004 (*F =* 3.25, *P =* 0.01; *W =* 0, *P =*0.007). These findings suggested that although canonical power bands were effective as kinematic biomarkers, using complex classifiers could offer a slight enhancement in movement prediction.^19^

**Figure 4:**
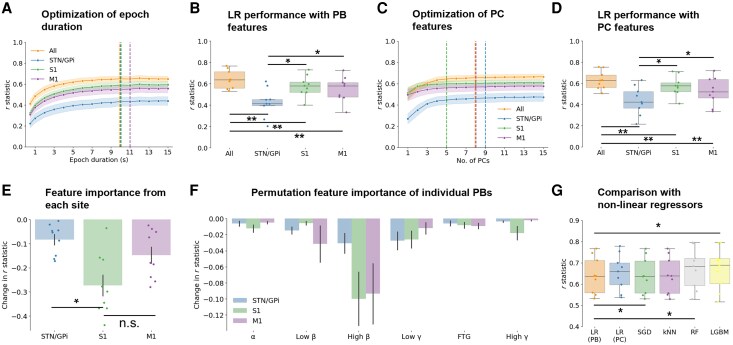
**Evaluating the performance of forearm movement speed regressors.** Linear regression (LR) models were trained to predict continuous forearm speeds. (**A**) The optimal epoch durations were identified for single-site (STN/GPi, S1 or M1) and combined regressors using Pearson’s *r* statistic values. (**B**) Evaluation of these LR models was performed on a holdout set. (**C**) Personalized features were developed using principal component analysis, and the optimal number of principal component (PC) features for each regressor was determined. (**D**) The performance of the personalized single-site and combined regressors with PC features was compared using the holdout set. (**E** and **F**) Permutation feature importance was computed for each brain region (**E**) and canonical power band (PB) (**F**). (**G**) Additional linear and non-linear models [Bayesian ridge regression (BR), stochastic gradient descent (SGD), *k*-nearest neighbours (KNN), random forests (RF) and light gradient boosted machines (LGBMs)] were developed using the canonical PB features. *P ≤ 0.05 and **P ≤ 0.01. FTG = finely tuned gamma; GPi = globus pallidus internus; M1 = primary motor cortex; S1 = primary somatosensory cortex; STN = subthalamic nucleus.

In addition to assessing binary movement states, we aimed to evaluate the utility of the multivariate signals in predicting absolute forearm speeds. To achieve this, we repeated our classifier analysis on linear regression models using canonical PBs from one or all sites and obtained similar results. Using Pearson’s correlations between predicted and actual speeds (*r* statistic) as the primary metric, we determined the optimal epoch duration for each model (STN/GPi, 10 s; S1, 10 s; M1, 11 s; combined, 10 s; Fig. 4A). Combining cortico-basal signals yielded the highest-performing models (mean ± SEM *r* statistic: 0.64 ± 0.03; *F =* 6.35, *P =* 0.002; *W =* 45.0, *P =*0.002; Fig. 4B). We also explored the use of personalized PCs as features. We identified the optimal number of PCs for each model (STN/GPi, nine; S1, five; M1, eight; combined, eight; Fig. 4C) and found that integrating PC features from all sites improved model performance (mean ± SEM *r* statistic: 0.63 ± 0.03; *F =* 4.69, *P =* 0.008; *W =* 45.0, *P =* 0.003; Fig. 4D), reinforcing the hypothesis that each site encodes distinct movement-related information.^52^

To assess the marginal movement decoding ability of each brain region, we calculated their respective permutation feature importance. Each site made significant, unique contributions to movement prediction (*W =* 0.0, *P =* 0.004) although S1 had higher feature importance than the STN/GPi (*W =* 44.0, *P =* 0.01; Fig. 4E). Cortical high beta power had the highest feature importance (mean ± SEM change in *r* statistic: −0.19 ± 0.03) compared with other PBs, including cortical low beta (*W =* 2.0, *P =* 0.006; Fig. 4F). This suggested significant differences between low and high cortical beta power in decoding forearm speed.^14^

As an additional measure of feature importance, we computed the conditional mutual information for each PB and brain region.^47-49^ Specifically, we calculated the marginal mutual information between each PB feature and forearm speed measurements, conditioned on the other cortical and subcortical PBs (Supplementary Fig. 5A). We also assessed the joint mutual information between PBs in each brain region and movement speeds, conditioned on PBs from other brain regions (Supplementary Fig. 5B). The conditional mutual information from the S1 was greater than that from the STN/GPi (*W =* 3.0, *P =* 0.029) but not significantly different from the M1 (*W =* 38.0, *P =* 0.056). Additionally, we found that cortical high beta power had the highest conditional mutual information (mean ± SEM MI: 0.020 ± 0.003). These findings corroborated our findings that the basal ganglia and sensorimotor cortex encode distinct, non-redundant movement-related information and that cortical high beta was the most predictive biomarker of naturalistic movement amongst the PBs (*F =* 2.67, *P =* 8.1 × 10^−4^; *W =* 2.0, *P =* 0.002).

Owing to the high multicollinearity among spectral features, canonical PBs were used for non-linear regression models (kNN, RF and LGBM). There was no significant variability in performance amongst the models (*F =* 0.28, *P =* 0.92; Fig. 4G). However, *post hoc* analysis revealed that the LGBM (mean ± SEM *r* statistic: 0.69 ± 0.03) and RF (mean ± SEM *r* statistic: 0.68 ± 0.03) regressors achieved significantly higher performance than the linear PB-based model (*W* > 1.0, *P <* 0.033). This further validated our finding that complex non-linear models could slightly improve model performance.^19,23^ For completeness, we re-ran our machine learning pipeline on the full patient cohort, including those with stimulation frequencies different from 130 Hz, and obtained similar results (Supplementary Table 1) for both the classifiers (Supplementary Fig. 3) and regressors (Supplementary Fig. 4).

### Effects of stimulation current amplitude on movement decoding

**Figure 5:**
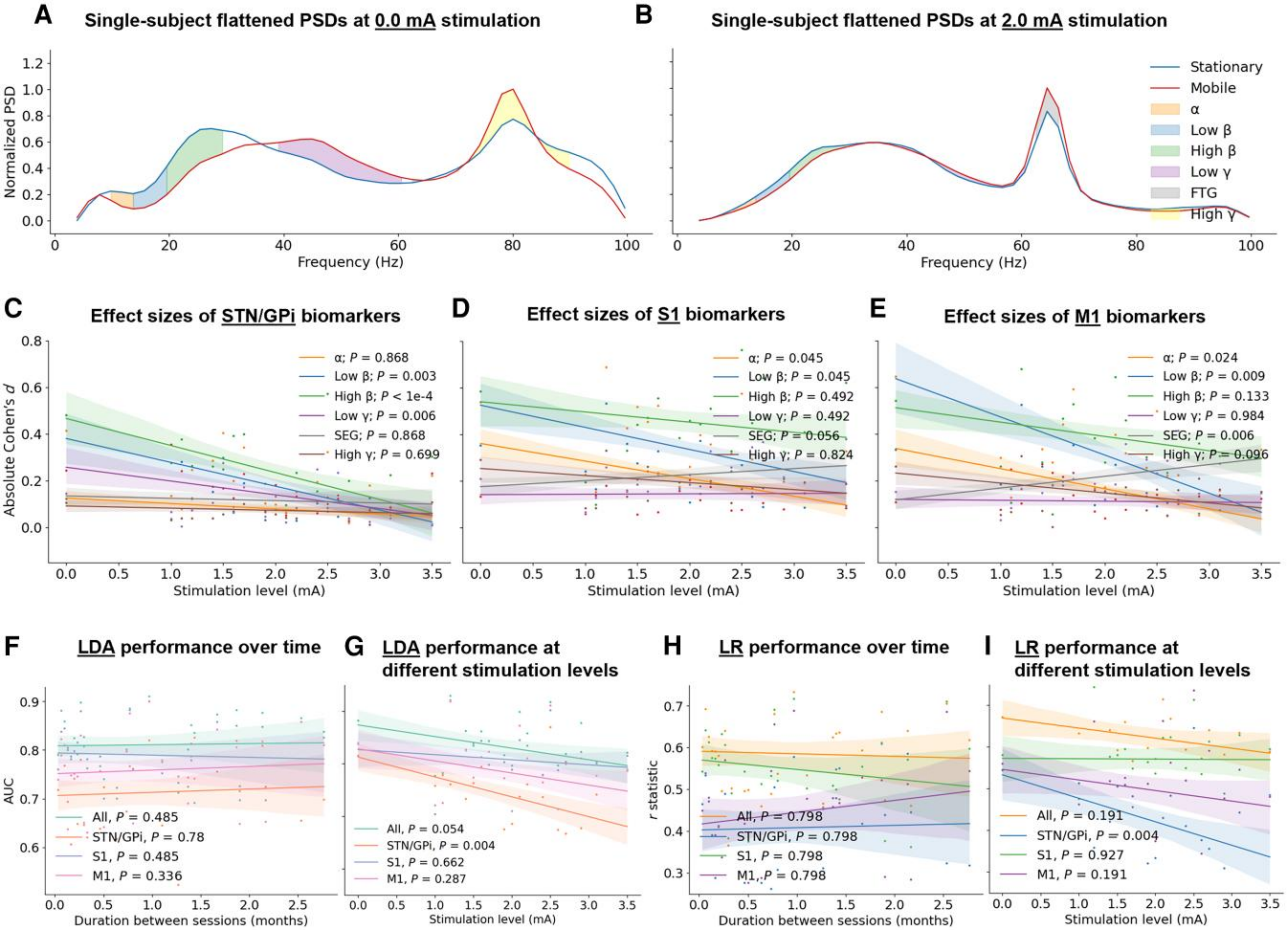
**Effects of stimulation on biomarkers of naturalistic movement.** (**A** and **B**) We computed the average normalized, flattened power spectral densities (PSDs) for the mobile and stationary states from the left subthalamic nucleus (STN) of a single subject at two stimulation levels: 0.0 mA (**A**) and 2.0 mA (**B**). The differences in spectral power across each canonical band are highlighted using different colours. We used linear mixed models (LMMs) to investigate how the absolute Cohen’s *d* effect size for each canonical power band varied across stimulation levels, which were treated as fixed effects. (**C**–**E**) To visualize these relationships, we plotted the average absolute Cohen’s *d* values across all patients for each stimulation amplitude in the following regions: STN/GPi (**C**), S1 (**D**) and M1 (**E**). (**F** and **G**) Additionally, linear mixed models (LMMs) were used to analyse variations in the area under the curve (AUC) for binary movement state classifiers, examining the effects of the duration between sessions (**F**) and stimulation level (**G**). (**H** and **I**) We also assessed changes in the *r* statistic for forearm movement speed regressors with respect to the duration between sessions (**H**) and stimulation level (**I**). In **F**–**I**, these relationships are illustrated by plotting the average model performance across all patients against either the duration between sessions or the stimulation level. For all analyses presented in **C**–**I**, individual patients and hemispheres were modelled as random effects in the respective LMMs. FTG = finely tuned gamma; Gpi = globus pallidus internus; LDA = linear discriminant analysis; LR = linear regression; M1 = primary motor cortex; S1 = primary somatosensory cortex.

Previous studies have demonstrated that DBS can influence neural signals, such as reducing resting-state subcortical beta activity in patients with Parkinson’s disease.^12,20,55^ However, the effects of varying stimulation levels on kinematic biomarkers and movement-predictive ML models have not been evaluated previously. Understanding this impact can also provide insights into the potential performance of movement-responsive BCI–aDBS systems, which decode neural signals in real time at different stimulation current amplitudes.^26,56^ To address this gap, we investigated the effects of stimulation levels on movement-related changes in alpha, beta and gamma PBs. An example of movement-related changes in STN spectral power for a single subject when DBS was at 0.0 or 2.0 mA is shown in Fig. 5A and B.

Absolute Cd effect sizes were used to quantify the movement-related desynchronization/synchronization of canonical PBs in each brain region. To evaluate the relationship between these effect sizes and stimulation amplitudes, we used LMMs, with stimulation amplitude modelled as a fixed effect, whereas individual patients and hemispheres were included as random effects. To visualize these relationships, we plotted the average absolute Cd values at each stimulation amplitude across all patients for the STN/GPi (Fig. 5C), S1 (Fig. 5D) and M1 (Fig. 5E). Increasing the stimulation amplitude was correlated with a decrease in the MRD of subcortical low beta [STN/GPi: *β* = −0.078; 95% confidence interval (CI) = (−0.1221, −0.036), *P =* 0.003], high beta [STN/GPi: *β* = −0.103; 95% CI = (−0.145, −0.060), *P <* 10^−4^] and low gamma [STN/GPi: *β* =−0.050; 95% CI = (−0.083, −0.018), *P =* 0.006], as evidenced by a reduction in absolute Cd values. In contrast, cortical regions showed no significant effect on high beta MRD. There was, however, a stimulation-related decrease in alpha MRD [S1: *β* = −0.034; 95% CI =(−0.061, −0.007), *P =* 0.045; and M1: *β* = −0.035; 95% CI = (−0.061, −0.008), *P =* 0.02] and low beta MRD [S1: *β* = −0.048; 95% CI =(−0.085, −0.010), *P =* 0.045; and M1: *β* = −0.072; 95% CI = (−0.118, −0.026), *P =* 0.009], in addition to an increase in M1 stimulation-entrained gamma MRS [M1: *β* = 0.044; 95% CI = (0.018, 0.069), *P =* 0.006]. This revealed that stimulation levels affected site-specific biomarkers of naturalistic motion.

To examine how the time interval between recording sessions affected the performance of movement-predictive classifiers, we trained the ML models on data from one session and tested them on data from subsequent sessions conducted at the same stimulation amplitude. We incorporated the duration between sessions as a fixed effect in our LMMs, while treating individual patients and hemispheres as random effects (Fig. 5F). Furthermore, we explored the impact of stimulation levels on classifier performance by performing 5-fold cross-validation on data collected from each session (Fig. 5G). We incorporated the stimulation level as a fixed effect in our LMMs, with individual patients and hemispheres treated as random effects. We then repeated this analysis to assess the effects of duration between recording sessions (Fig. 5H) and stimulation amplitude (Fig. 5I) on regressor performance. The performance of both the classifiers and regressors maintained stability over time (maximum duration, 83 days; median ± SEM duration, 6 ± 2 days; *β* < 0.033, *P >* 0.34). STN/GPi classifiers [*β* = −0.030; 95% CI = (−0.046, −0.013), *P =* 0.004] and regressors [*β* = −0.054; 95% CI = (−0.086, −0.023), *P =* 0.004] demonstrated lower performance at higher stimulation amplitudes. Conversely, the S1, M1 and combined models were not significantly affected by changes in stimulation levels (*β* > −0.019, *P >* 0.05). This was probably attributable to the preserved predictive power of cortical high beta MRD at higher stimulation amplitudes.

## Discussion

We identified cortico-basal biomarkers of naturalistic forearm movement by analysing >530 h of neural and accelerometry recordings from 15 patients with Parkinson’s disease during DBS.^21,44^ Specifically, we observed beta MRD and gamma MRS in both cortical and subcortical regions.^13,50,51,57^ Although we did not track the medication timings of our patient cohort specifically, we repeated our biomarker analyses within 15 min windows, during which medication states were likely to be stable. This approach demonstrated that these biomarkers remained predictive of movement across different medication states, rather than merely decoding slow medication state fluctuations. Taking a multivariate ML approach, we revealed distinct contributions from each brain region in predicting movement, with cortical high beta MRD being the most discriminative signal.^52,58^ Consequently, ML models that incorporated both cortical and subcortical signals demonstrated superior model performance in comparison to single-site models. We also found that DBS amplitudes influenced the performance of these models, with higher stimulation levels leading to a decrease in the accuracy of subcortical models.^26,55^ Despite this, cortical and combined cortico-basal models continued to perform well at higher stimulation levels. This sustained performance supports the future potential of movement-responsive BCI–aDBS.^23,24^

### Subcortical low and high beta power encode distinct movement-related functions

Within the beta frequency range (12–30 Hz), we revealed group-level functional distinctions between low (12–20 Hz) and high (20–30 Hz) subcortical beta rhythms, which have both been associated previously with motor execution.^13^ Higher Parkinson’s disease-related severity of bradykinesia has also been correlated with increased low beta power.^12^ Treatments such as dopaminergic medication and DBS have been found to suppress low beta activity and reduce bradykinesia severity.^58,59^ In contrast, high beta has been linked to physiological mechanisms, such as force generation and voluntary motor actions.^60^ This suggests that low beta might be functionally ‘anti-kinetic’ in the pathophysiology of Parkinson’s disease, whereas high beta might have a stronger association with the execution of normal motor plans.^14^ Our study supports this difference in physiological roles by demonstrating that MRD associated with naturalistic movement, irrespective of the dopaminergic state, was observed only within the high beta band.

### Cortical stimulation-entrained gamma is associated with naturalistic movement

Cortical and subcortical broadband gamma MRS has been linked to motor speed and complexity in both healthy individuals and patients with Parkinson’s disease.^50,57^ During DBS, cortical and subcortical oscillations at subharmonics of the stimulation frequency have also been observed.^21^ Specifically, entrainment at the half-harmonic (∼65 Hz) of the 130 Hz stimulation frequency has been noted.^61^ Broadband gamma activity is non-oscillatory and thought to represent asynchronous neuronal spiking activity across a wide frequency range (30–200 Hz).^62^ In contrast, recent studies have shown that stimulation-entrained gamma oscillations are associated with synchronized spiking and are influenced by dopaminergic medication and sleep–wake cycles, indicating a non-artefactual, physiological role for these neural signals.^30,31^ Consistent with these findings, we detected broadband gamma movement-related synchronization within the cortical regions. In our cohort of patients stimulated at 130 Hz, stimulation-entrained gamma MRS at ∼65 Hz was significantly greater than that at the other frequencies within the broadband gamma range.^63^ This suggests that entrained gamma power distinguishes more effectively between movement states than broadband gamma and might serve as a valuable kinematic biomarker in the ON-stimulation state.^64^

### Subcortical and cortical sites provide complementary information for movement decoding

The sensorimotor cortex is involved in motor planning and execution, whereas the basal ganglia play a more implicit, regulatory role in selecting and initiating movements, inhibiting unwanted actions and refining motor skill.^2,4,8,9^ Furthermore, prior research by our group revealed that combining spectral features from cortical and subcortical regions can improve the accuracy of linear models in decoding Parkinson’s disease-related symptom states, such as severe bradykinesia or dyskinesia.^22^ These findings suggest that cortical and subcortical signals encode specific information regarding naturalistic motion. Consistent with these findings, this study demonstrates that integrating subcortical and cortical signals yielded more accurate ML models for predicting movement than using signals from individual sites alone, indicating complementary encoding. Feature importance analyses further confirmed the significant contributions of each brain region to the performance of the combined models. Repeating our analysis on the full patient cohort, we observed similar model performance and feature importance across different stimulation frequencies (other than 130 Hz).

### DBS modulates movement-related cortico-basal biomarkers

Movement-related changes in the spectral power of cortical and subcortical biomarkers were influenced by DBS amplitudes. Specifically, increased stimulation levels were found to be correlated with lower subcortical beta MRD and gamma MRS, as evidenced by a reduction in absolute Cd effect size.^26^ This reduction potentially impacted the discriminative power of beta and gamma biomarkers at higher stimulation levels, thus decreasing the accuracy of subcortical models for movement prediction. In the sensorimotor cortex, higher DBS levels were associated with reduced low beta MRD, suggesting that patients could achieve similar movement speeds with decreased beta MRD. This aligns with previous findings showing higher cortical beta MRD in Parkinson’s disease patients than in individuals with essential tremor or without movement disorders.^17,65^ Moreover, dopaminergic medication is associated with a reduction in cortical beta MRD and an improvement in motor speed.^51^ In contrast to our subcortical models, those using cortical signals or integrating cortico-basal signals demonstrated greater stability. This was likely to be attributable to the resilience of cortical high beta MRD, identified as the most discriminative kinematic biomarker. Therefore, integrating cortico-basal signals could enhance the accuracy and stability of movement prediction across varying stimulation conditions, which is crucial for the development of BCI–aDBS systems to treat motor disorders.

### Implications for closed-loop DBS treatments

Based on the gating theory of basal ganglia function, the basal ganglia excessively inhibit the initiation and execution of motor plans, exacerbating these hypokinetic symptoms.^25^ By increasing stimulation amplitudes during movement, we might be able to disinhibit the execution of these movements only when it is most needed and alleviate bradykinesia severity.^24^ However, ensuring the therapeutic efficacy of a movement-based adaptive DBS policy requires careful optimization of both upper and lower stimulation levels. The upper stimulation levels must be constrained to prevent excessive stimulation, which can lead to side effects such as dyskinesia, particularly during medication-ON periods. Likewise, the lower stimulation levels should not be set too low during rest periods, to avoid breakthrough tremor, especially during medication-OFF periods.

To be deployable for everyday use, such an adaptive DBS paradigm must be able accurately to decode unconstrained, volitional motion from neural signals. However, previous studies have primarily focused on decoding movement during short, constrained tasks, such as finger-tapping and hand rotations. Our study is the first to demonstrate the accurate prediction of unsupervised, naturalistic movements using chronic neural recordings during DBS (classifier AUC > 0.85 and regressor *r* statistic > 0.68). We also highlight the importance of recording neural signals from both cortical regions and the basal ganglia, because integrating these signals might improve the performance and stability of movement-predictive models. With these findings, we support the development of an integrated BCI–aDBS approach that combines the fast and precise motor-decoding capabilities of emerging BCIs with the therapeutic neuromodulatory effects of aDBS.

## Conclusion

Our findings demonstrate the complimentary contributions of cortico-basal neural circuits and signals in naturalistic, unconstrained movements. We identified multivariate movement encoding particularly through cortico-basal beta and gamma oscillations, in addition to stimulation-entrained gamma, in the presence of DBS. We developed ML techniques for fast and accurate decoding of unconstrained movement and identified features that were resilient to changes in stimulation conditions. This validates the potential feasibility of BCI–aDBS in naturalistic settings and opens a translational pipeline for precise motor network re-tuning in disorders of movement, including Parkinson’s disease and stroke.

## Supplementary Material

awaf466_Supplementary_Data

## Data Availability

De-identified, processed neural and accelerometry data can be provided upon request according to the data-sharing policies of the National Institutes of Health (NIH).
