# Supplementary material for: Cortico-basal oscillations index naturalistic movements during deep brain stimulation
Source: Brain. 2025 Dec 16;149(8):2702–15. doi: 10.1093/brain/awaf466 (PMC13431667; doi:10.1093/brain/awaf466)
Supplement: awaf466_Supplementary_Data [file awaf466_supplementary_data.pdf]

Supplementary Material

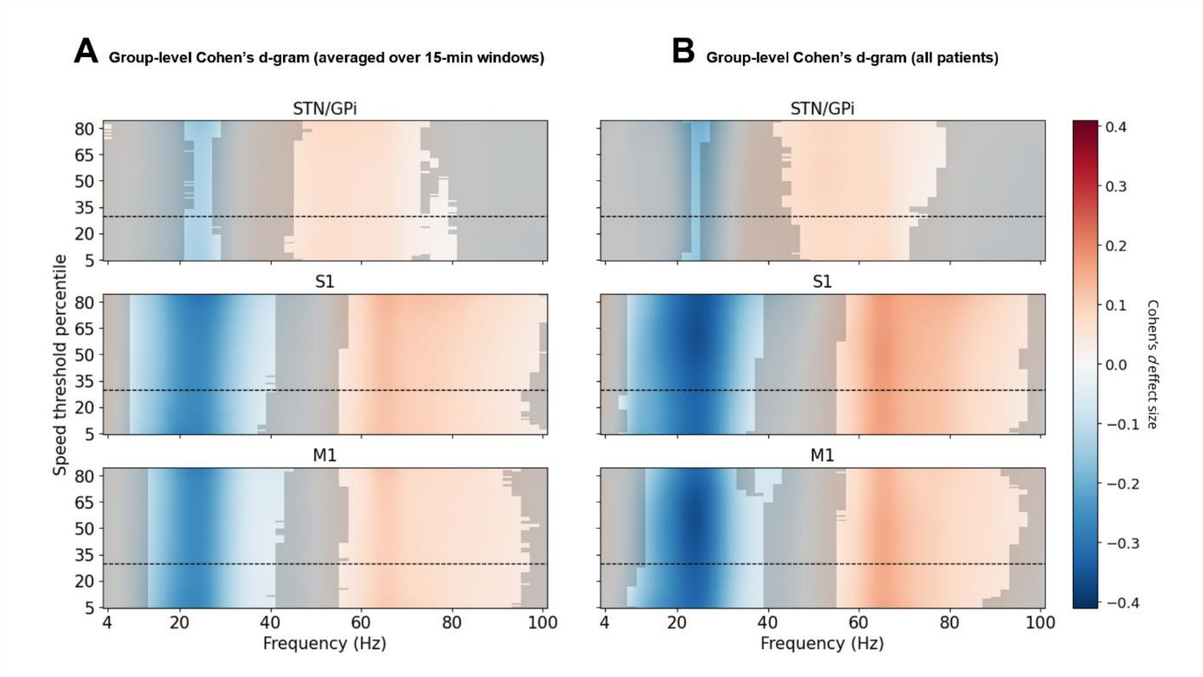

Figure S1 (A) To assess the stability of the kinematic biomarkers over short time windows, we computed Cohen's d-grams within 15-minute intervals and averaged them for each patient and hemisphere. (B) We also produced the group-level Cohen's d-gram for the full recording duration without excluding periods of tremor or dyskinesia, as detected by the Apple watch algorithms. In both plots, we observed FDR-corrected group-level MRD and MRS bands within each brain region that were consistent with those in Figure 2B. The grey mask indicates the regions that did not survive FDR-correction ($P > 0.05$).

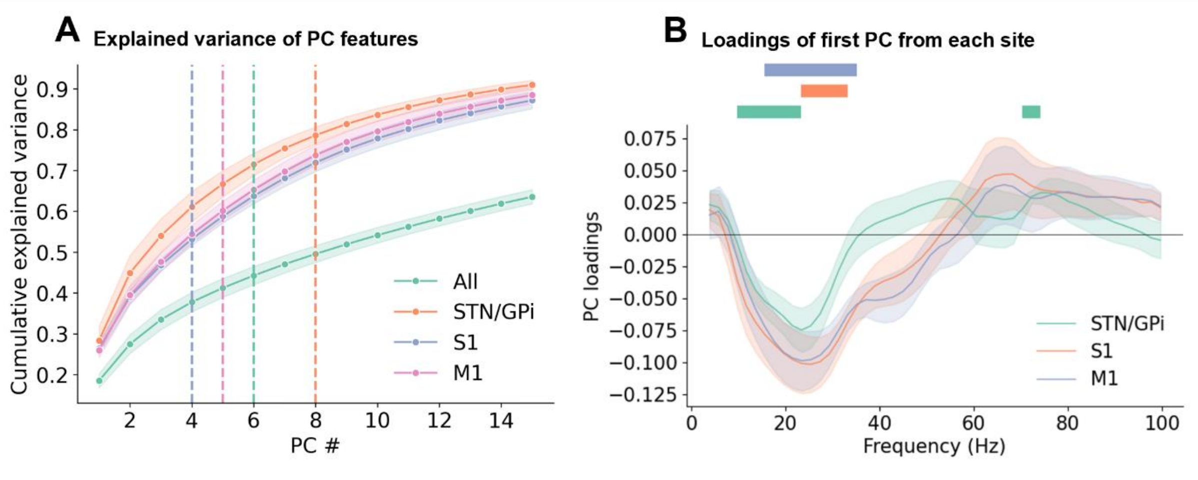

Figure S2 Principal component analysis (PCA) was performed on the power spectral densities (PSDs) from each brain region or from combining signals from all regions. (A) The cumulative explained variance for the resulting PCs were computed. (B) The first PC from each site explains the most variance and the respective loadings were identified for each patient. The average PC loadings and the statistically significant frequency bands after FDR-correction ($P < 0.05$) were plotted.

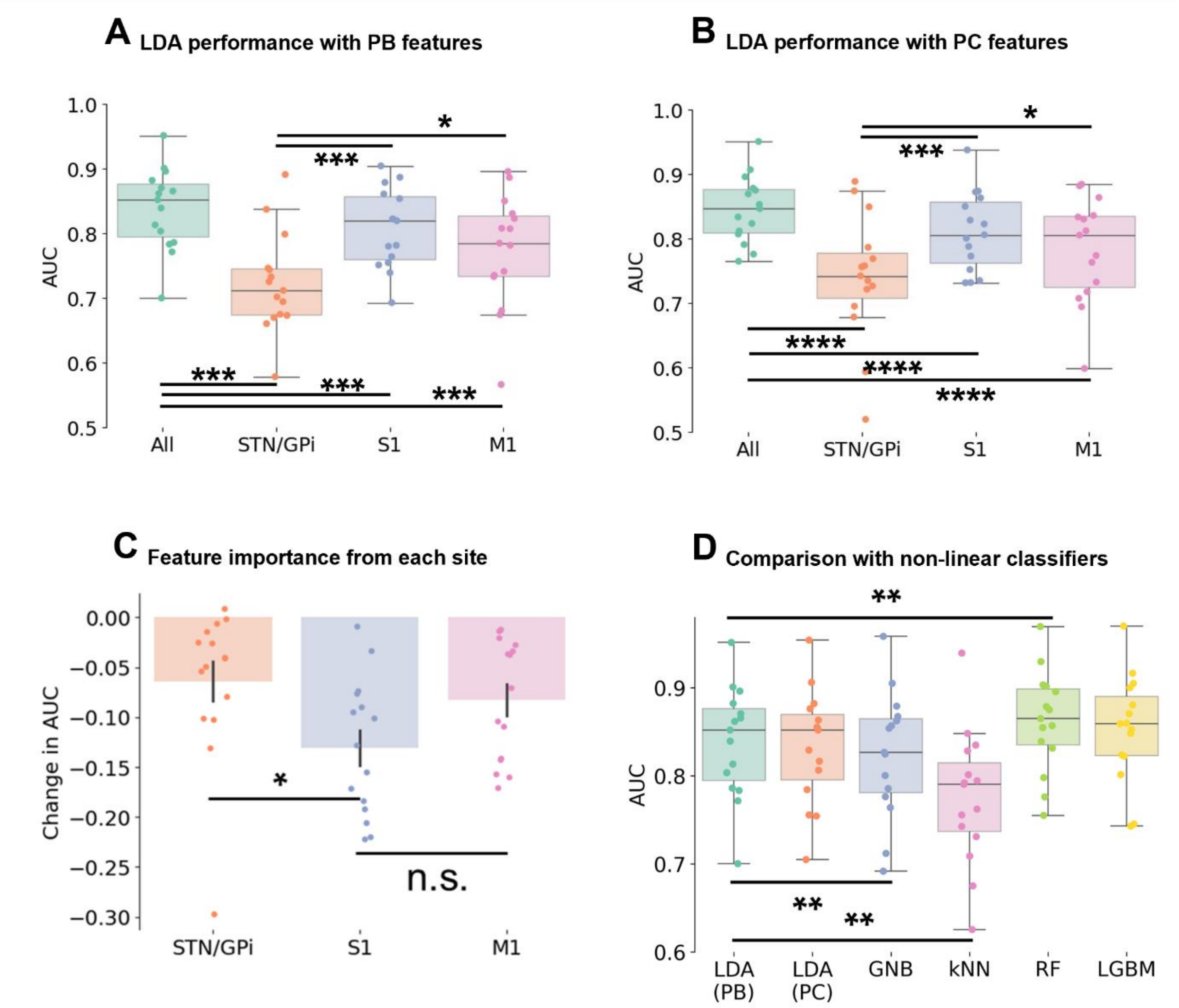

Figure S3 The neural signals from the full patient cohort, including those who were OFF stimulation or had stimulation frequencies other than 130 Hz, were processed and binary movement state classifiers were developed. The use of (A) power band (PB) and (B) PC features for these Linear Discriminant Analysis (LDA) classifiers was evaluated on a holdout set. (C) The permutation feature importance of signals from each site was computed. (D) Non-linear models were developed using all PSDs as features to compare their performance with linear models that used PB or PC features. $*P \leq 0.05$, $**P \leq 0.01$, $***P \leq 0.001$, $****P \leq 10^{-4}$.

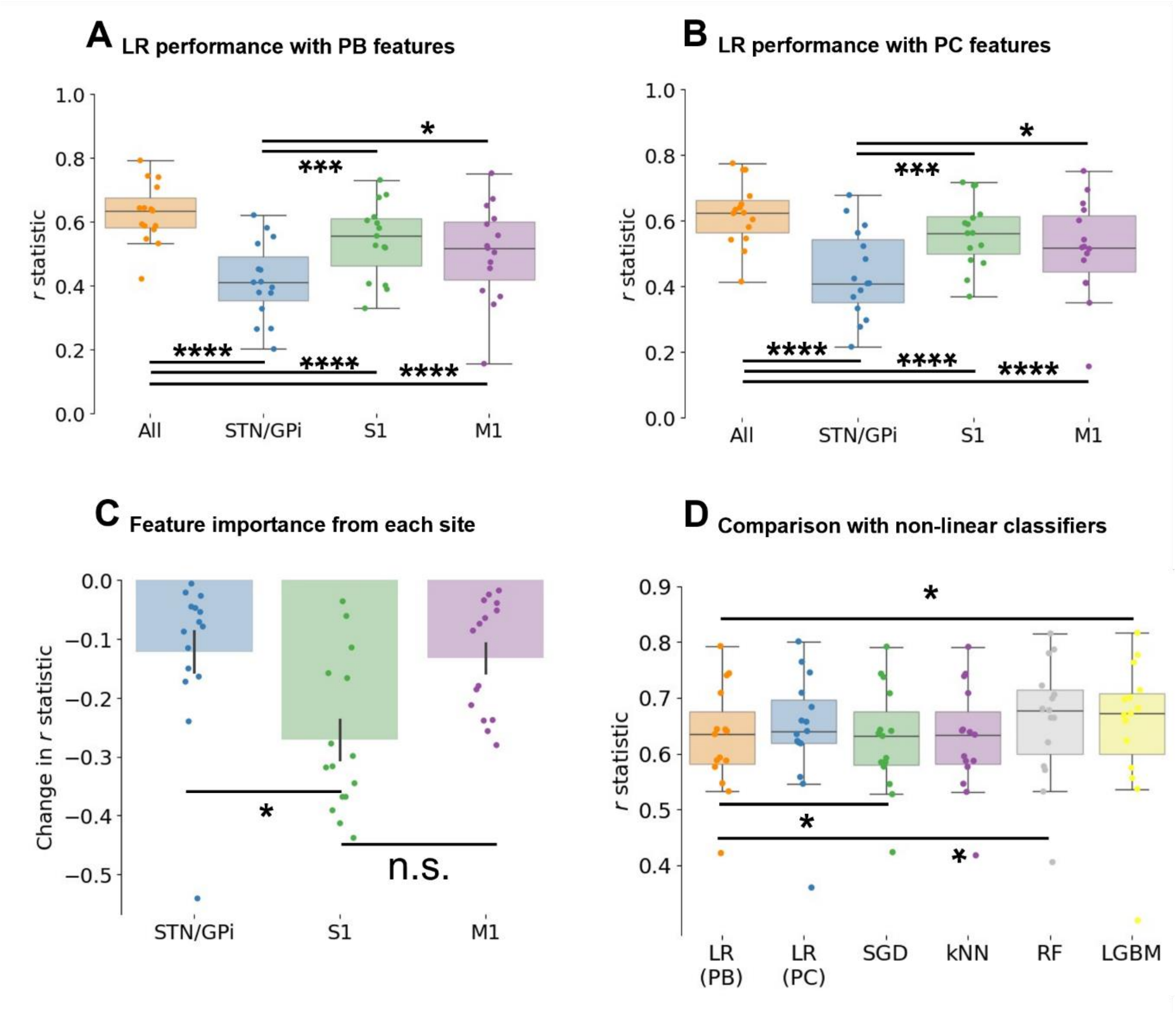

Figure S4 Linear regression (LR) models to predict continuous forearm speed were developed for the full patient cohort and the resulting *r* statistic values were calculated when using (A) PB and (B) PC features. (C) The feature importance of biomarkers from each site was also determined for these linear regressors. (D) Non-linear regressors were also explored and performance of these models were computed for comparison. *$P \leq 0.05$, **$P \leq 0.01$, ***$P \leq 0.001$, ****$P \leq 10^{-4}$.

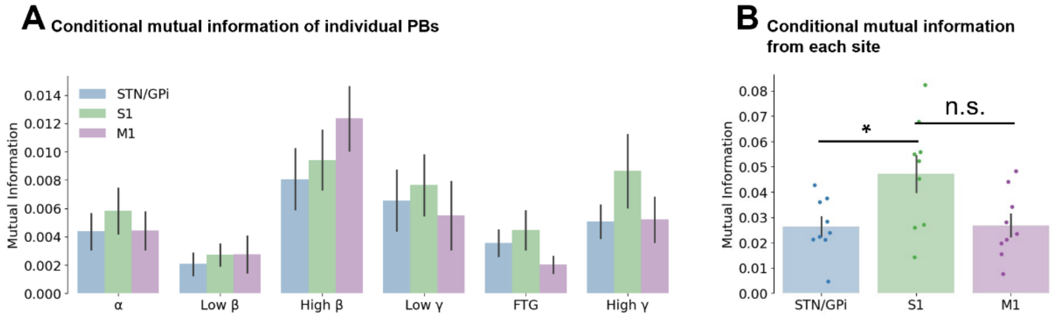

Figure S5 (A) Marginal mutual information between each canonical power band (PB) feature and continuous movement speeds, conditioned on the other PBs. (B) Joint mutual information between PB features from each brain region and movement speeds, conditioned on the PBs from the other brain regions. *$P \leq 0.05$, **$P \leq 0.01$, ***$P \leq 0.001$, ****$P \leq 10^{-4}$.

Table S1 Average performance of linear models trained on full patient cohort

Metric	Combined	STN/GPi	S1	M1
**Mean ± SEM Classifier Performance**				
**AUC**	0.84 ± 0.02	0.72 ± 0.02	0.81 ± 0.02	0.77 ± 0.02
**Balanced Accuracy**	0.77 ± 0.01	0.67 ± 0.02	0.74 ± 0.01	0.71 ± 0.02
**F1 Score**	0.81 ± 0.02	0.72 ± 0.02	0.78 ± 0.02	0.76 ± 0.02
**PPV**	0.86 ± 0.02	0.79 ± 0.04	0.85 ± 0.03	0.83 ± 0.03
**Sensitivity**	0.77 ± 0.02	0.68 ± 0.02	0.74 ± 0.02	0.72 ± 0.02
**Specificity**	0.77 ± 0.02	0.66 ± 0.02	0.74 ± 0.01	0.70 ± 0.02
**Mean ± SEM Regressor Performance**				
**r statistic**	0.63 ± 0.02	0.41 ± 0.03	0.56 ± 0.03	0.52 ± 0.04
**MSE**	0.41 ± 0.02	0.57 ± 0.03	0.48 ± 0.03	0.49 ± 0.03

SEM: standard error of the mean, AUC: area under receiver operating characteristic curve, PPV: positive predictive value, r statistic: Pearson correlation coefficient between true and predicted values, MSE: mean squared error.
